# Acting within an increasingly confined space: A qualitative study of sexual behaviours and healthcare needs among men who have sex with men in a provincial Tanzanian city

**DOI:** 10.1371/journal.pone.0183265

**Published:** 2017-08-17

**Authors:** Markus Larsson, Jasmine Mohamed Shio, Michael W. Ross, Anette Agardh

**Affiliations:** 1 Division of Social Medicine and Global Health, Department of Clinical Sciences Malmö, Lund University, Malmö, Sweden; 2 Amsterdam Institute for Social Science Research, Department of Anthropology, University of Amsterdam, Amsterdam, The Netherlands; 3 Programme in Human Sexuality, Department of Family Medicine and Community Health, University of Minnesota, Minneapolis, Minnesota, United States of America; David Geffen School of Medicine at UCLA, UNITED STATES

## Abstract

**Objective:**

To explore risk perceptions, sexual practices and healthcare needs among men who have sex with men in the provincial city of Tanga in northern Tanzania. Previous research suggests that HIV/STIs are increasing problems for this population. Yet, few studies have been conducted outside the urban area of Dar es Salaam, which has limited our knowledge about the HIV/STI risk factors and healthcare needs among men who have sex with men who live outside major metropolitan areas.

**Method:**

During three months in 2013, 10 in-depth interviews with men who have sex with men were conducted in Tanga. Data were interpreted through qualitative content analysis.

**Results:**

The theme that emerged was labelled “Acting within an increasingly confined space”. The theme reflects the interference of stigma in men’s lives, and in the face of potential discrimination, men perceived their sexual and healthcare choices as limited. This created obstacles for forming romantic and sexual relationships, insisting on consistent condom use with sexual partners, maintaining open and conducive relationships with family, and accessing healthcare services when required.

**Conclusions:**

Sexual stigma is a concern as it contributes to HIV/STI risk-related behaviours among men who have sex with men. Priority should be given to programmes that support same-sex practicing men in their efforts to make informed choices regarding their sexual health. Creating safe cyber networks provides an opportunity to reach this population with targeted sexual health education messages. Such programmes might be even more urgent in smaller towns and rural areas where gay specific initiatives are more limited than in urban areas.

## Introduction

Previous studies from Tanzania have demonstrated that men who have sex with men (MSM) suffer from high levels of HIV and STI infection [1, 2]. Furthermore, qualitative studies have revealed that MSM face stigma based on sexual orientation/behaviour when seeking healthcare services, which has created barriers to timely diagnosis and treatment [3, 4]. At the same time, research has shown that sexual risk perceptions and preventive measures might be low among MSM, and high levels of commercial sex work, condomless sex, substance abuse, and depression have been reported [5–8].

There is currently a rich body of literature from Sub-Saharan Africa showing increased risk of HIV/AIDS among MSM in urban communities, but research regarding risks, risk perceptions, and sexual behaviour among MSM who live beyond larger cities remains scarce [9–17]. Wade and colleagues demonstrated already in 2005 that MSM were at high risk for HIV infection in Senegal with a prevalence of 21.5% of the 463 surveyed [18]. Sanders et al. in their study among 285 MSM in Mombasa, Kenya, found that 43% of MSM who exclusively had sex with other men were HIV positive and among those MSM who reported sex with both men and women, HIV prevalence was 12.3% [19]. Since then, studies have established that Sub-Saharan African MSM, as in other parts of the world, are at higher risk of HIV infection compared to the general population [20, 21]. In the context of Tanzania, a bio-behavioural study among 753 MSM in Dar es Salaam showed that the overall HIV prevalence was 22.2% [2] and a consensus size estimate of 49 000 MSM living in the urban areas of mainland Tanzania projected a prevalence of 25% [22]. A complex web of limited knowledge and erroneous beliefs, high-risk sexual practices and in particular condomless receptive anal sex, substance abuse, and stigma appear to be the main drivers of the epidemic.

The aim of this qualitative study was to explore risk perceptions, sexual practices, and healthcare needs among MSM in the provincial city of Tanga in northern Tanzania. Specifically, the study sought to understand the factors that contribute to HIV/STI risks in this MSM population residing far from the metropolitan city of Dar es Salaam and outside mainstream gay venues. While the criminalisation of homosexuality in the majority of Sub-Saharan African countries most certainly affects the development of a “gay scene”, areas outside of major metropolitan centres generally attract fewer LGBT (lesbian, gay, bisexual and transgender) organisations and prevention programmes [17]. This could potentially influence how MSM perceive and relate to HIV/STI risks. A qualitative study in rural South Africa previously revealed that MSM lacked HIV knowledge about male-to-male sex and had difficulties in negotiating condom use with their partners [23]. Thus, the findings from the current study could provide further information as to whether specially targeted interventions are required for Sub-Saharan African MSM who reside outside of major urban centres.

## Method

### Study setting

The provincial city of Tanga is a northerly coastal city in Tanzania located in Tanga region. The city had in 2012 a population of approximately 273 000 [24]. While it is one of the larger cities, its average annual population growth rate between 2002 and 2012 was 2.2%, which is below the national average of 2.7% [24]. Very few studies have been conducted on MSM in Tanga. A report on the LGBT situation in Tanzania described Tanga as having a long cultural tradition of including gay men in community life if they refrain from openly revealing their sexual orientation [25]. They are, for example, invited to entertain at social functions such as weddings. The report further asserted that gay men in Tanga tend to form networks with female sex workers in order to gain friendship and access to social support [25]. In a study by Ross et al. of 100 MSM in Tanga, 76.8% reported stigma towards gay men among the general community and 67.7% among doctors and nurses [26]. Ross et al. further found that 11.3% were HIV positive and 4.4% had a curable STI [1]. Also, an assessment of social networks found that the mean reported personal network size of the 100 MSM in Tanga was 7.6 [26]. This suggests that there are existing networks of MSM in Tanga despite that homosexual behaviours are illegal in Tanzania [27]. Albeit most LGBT organisations are centralised to Dar es Salaam, there is at least one organisation in Tanga that works with HIV/AIDS advocacy and economic empowerment for the LGBT group [28].

### Data collection

The study was nested within a larger cross-sectional study of 100 MSM in Tanga 2013 [1]. The study used a respondent-driven sampling (RDS) technique to recruit participants to investigate HIV/STI prevalence, sexual history and sexual and social networks. A detailed description of the sampling frames has previously been presented [1, 26]. Study participants had to be at least 18 years of age and should have reported sexual relations with another man during the last six months. After recruitment to the study, survey participants were referred to a private house rented for the project where data collection took place. To recruit participants in an unbiased manner for the qualitative study, every 10^th^ participant in the quantitative study was asked if he wanted to participate in a qualitative study about his relationships, sexual practices, social and family networks, and healthcare-seeking behaviours. No man declined participation and the total sample of participants was therefore ten.

After agreeing to participate in an interview, the men were asked to suggest a place for the interview that was convenient for them. Before the interview began, we obtained permission to audio-record the interview, and informed consent was given after reading the letter of consent aloud. One of the co-authors (JS) is a native speaker and was thus responsible for conducting the interviews in Swahili, which is spoken throughout Tanzania. The men were informed that participation was anonymous, voluntary, and that they could refuse participation or withdraw from the interview at any time. After the participants had given verbal consent (all participants gave their verbal consent), an interview was conducted. To gain access to in-depth information a thematic interview guide was developed (see S1 and S2 for the interview guide). This guide was used as a framework to cover the topics of interest [29]. The open-ended questions were intended to explore the men’s views, perceptions and experiences regarding their intimate and sexual life, stigma and discrimination, and healthcare needs. Participants were asked questions and encouraged to freely reflect in their responses. The interviews lasted for approximately one hour. Due to the fact that same sex relationships are criminalised in Tanzania, no names, contact details or physical information were taken that could be used to trace the participants. The interviews were translated into English by a professional translation bureau and cross-checked for accuracy by the same person (JS) who conducted the interviews. The data collection took three months in total (from January to March 2013).

Ethical permission for the study was obtained from the Tanzanian National Institute for Medical Research (NIMR/HQ/ R.8a/Vol. IX/1088) and the University of Texas Health Science Center’s Institutional Review Board (HSC-SPH-10-0033).

### Data analysis

In order to fully grasp the rich information that emanated from the in-depth interviews, a manifest and latent qualitative content analysis was employed, according to the method suggested by Graneheim and Lundman [30]. First, after reading the interviews extensively, the first author (ML) coded the data using MAXQDA 12 (VERBI Software, Berlin, Germany), a software programme for qualitative data analysis. After the data were condensed into smaller units of analysis, codes that labelled their meanings were assigned [30]. Once the first author had finalised the coding template, he shared it with the other co-authors to obtain their feedback regarding accuracy and consistency. Next, codes that expressed similar contents were clustered to form categories. Thus, the categories represent the manifest content and were grounded in the words of participants–either as direct quotes or reflecting the commonality of a group of codes [30, 31]. The co-authors constantly compared the codes that had been grouped under one category with codes that belonged to another category to ensure that the categories accurately described the phenomenon they represented [32]. Finally, categories were examined for the identification of emergent themes that linked the underlying meanings across the categories. This step of the analysis represents the latent part, as it attempts to describe the underlying essence of the categories on an interpretive level [30]. The co-authors conjointly compared similarities and differences across the categories and in this manner identified one theme and four sub-themes.

## Findings

**Fig 1:**
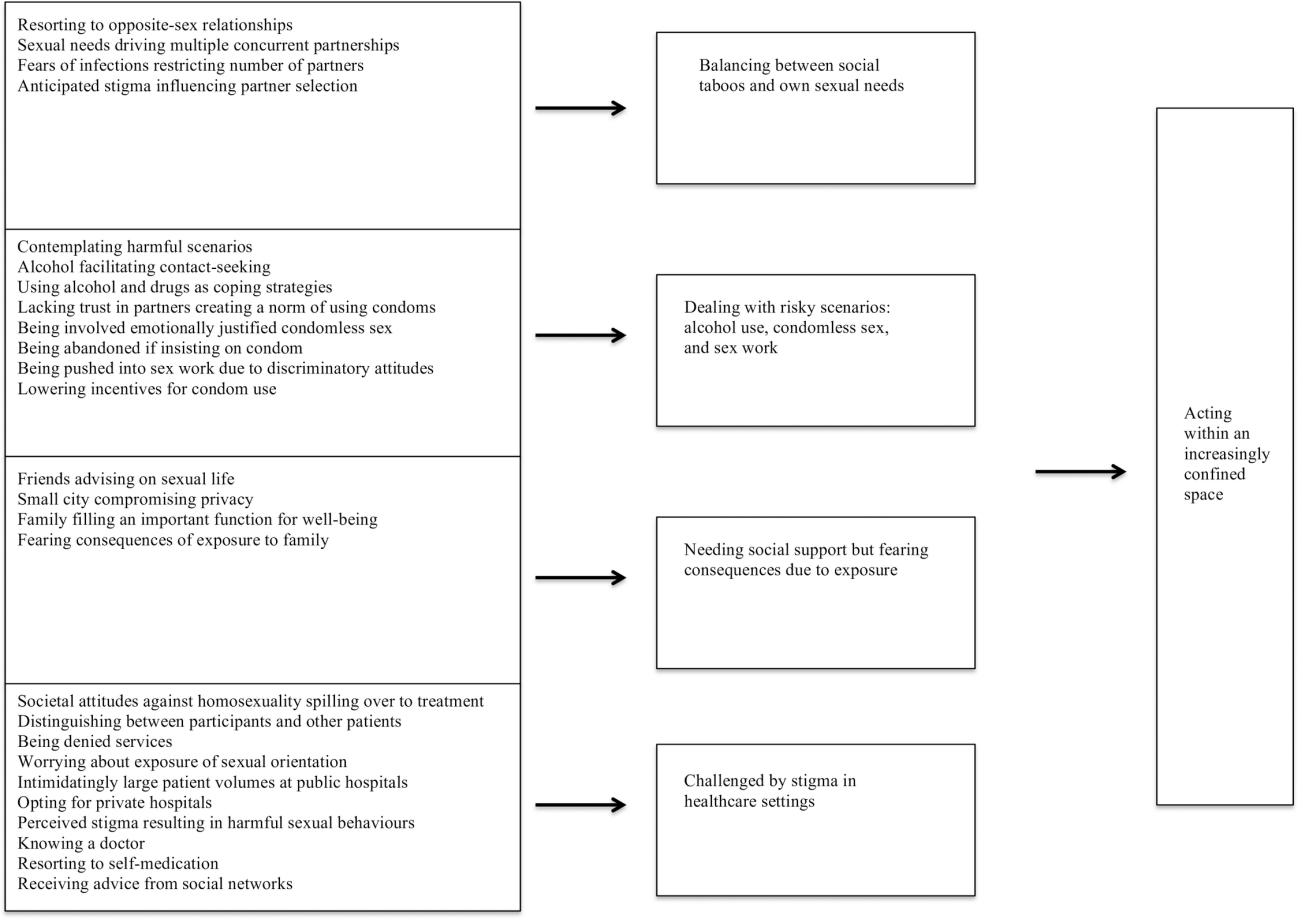
Analytical model of MSM’s risk perceptions, sexual practices and healthcare needs. Categories, sub-themes and theme (from left to right).

The analysis of the data showed that participants struggled with the tensions created by social norms opposing homosexuality and their desires for other men as well as needs to take care of their health. The theme that emerged, ‘Acting within an increasingly confined space’, represents the experiences men had of stigma and how these affected their attitudes, perceptions and behaviours related to identity, relations and lifestyle choices. The theme depicts how the omnipresence of the heterosexual norm in men’s surroundings constructed a confined space in which men’s options and alternatives diminished in fear of being further ostracised and isolated. Yet, MSM tried to reclaim their agency through strategies that had been developed in order to cope with the social control that the family and community exerted over them. These strategies enabled men to be intimate, engage in sexual relationships and receive social support from likeminded peers. As shown in Fig 1, the theme consists of four sub-themes: ‘Balancing between social taboos and own sexual needs’, ‘Dealing with risky scenarios: alcohol use, condomless sex, and sex work’, ‘Needing social support but fearing consequences due to exposure’ and ‘Challenged by stigma in healthcare settings’. Each sub-theme is built upon a number of categories (underlined within single quotation marks in the text) grounded in the words of participants, which work in tandem to illustrate the men’s perceptions and experiences.

### Balancing between social taboos and own sexual needs

The types and forms of sexual relationships that participants engaged in varied considerably. Sexual activity was influenced by the strict social taboo against homosexuality and participants appeared to manage this by concealing sexual relationships. The story of “Ibrahim” was a typical example. During adolescence Ibrahim struggled with homoerotic feelings and had problems with acknowledging these desires due to the anti-homosexual attitudes of his family and community. He has tried occasional celibacy and to date women but says his urges for men are too strong. Currently, he has two secret male lovers that he sees monthly.

During the interviews, participants referred to themselves as gay, using words such as “gei”, “ghei” or the plural “magei”, which are local expressions for the English word gay. Yet, ‘resorting to opposite-sex relationships’ was seen as a necessary strategy to adhere to sexual norms:

“I like male sexual partners more but in most cases I keep girlfriends around so that people I know won’t find out that I am gay. I like to walk with them in the streets for show off so that people see me dating a woman. This helps me to hide my true identity.” (Interview 5)

Participants described feelings of being seen through the narrow lens of heterosexuality, and felt pressured to make lifestyle choices that adhered to social expectations: “If people don’t see me dating a woman they will know that I’m gay” (Interview 1). To engage in heterosexual interactions and have these noticed by the community was therefore considered as critical by MSM in order to avoid suspicions about their sexual orientation.

The category ‘sexual needs driving multiple concurrent partnerships’ describes how participants dealt with sexual desires and attraction to other men:

“I have one permanent partner. This partner isn’t in town at the moment so I have another casual partner whom I can call anytime whenever I want to have sex with a man.” (Interview 3)

MSM stated that they intentionally developed and fostered multiple sexual partnerships to secure the availability of sexual options to be able to fulfil their sexual and intimate needs: “I have ever had five boyfriends, I’m still dating four of them, and can always call one of them if the other is busy” (Interview 7). However, a general sense of anxiety was perceived in having multiple sex partners, and ‘fears of infections restricting number of partners’ were also mentioned. Participants worried about the chance of being exposed to various infections, which acted as a barrier for multiple and concurrent partnerships:

“I’m so scared of getting diseases so I have one boyfriend only. If I keep many sexual partners then it will be easy for me to get sick and this will hurt me. At least with my current boyfriend I know that I won’t get sick from having others.” (Interview 10)

‘Anticipated stigma influencing partner selection’ was mentioned by men who were single, which created challenges for forming relationships:

“I can fall in love with a man but I can’t tell him that I want to be with him because of the fear that he may abuse me or even tell my family.” (Interview 8)

The fear seemed to be closely related to the conservative climate around homosexuality. Thus, concerns of potential repercussions from revealing own sexual orientation limited men’s potential to identify partners. However, there were also other reasons mentioned for not wanting or being able to form same-sex relationships. These were mainly issues related to practicalities such as “I can’t take a boyfriend home” (Interview 5) or trust such as “[it is] impossible to know if he’s faithful” (Interview 1).

### Dealing with risky scenarios: Alcohol use, condomless sex, and sex work

Alcohol use, condom use and commercial sex work emerged as critical dimensions of men’s sexual behaviours. These were influenced by own personal knowledge and attitudes, desires and fear as well as stigma and discrimination. When discussing stigma, a general sense of isolation or rejection was noted. Men often used “unyanyapaa” or “ubaguzi”, which in English mean discrimination or segregation when describing negative scenarios related to their sexual orientation. Participants exemplified with people who stopped offering support, being fired from a job, or not being involved in family decisions. Altogether, this contributed to reduced self-esteem and dignity, loneliness, and mistrust towards other people.

Men in this study were well informed about the risks associated with alcohol and sex and ‘contemplating harmful scenarios’ that could expose them to HIV infection was common:

“I know that alcohol use is a risky behaviour and it can expose me to HIV. That’s why I rarely drink. Whenever I take alcohol I don’t usually let myself be unconscious because I can end up practicing unprotected sex and get HIV.” (Interview 1)

Concerns that alcohol could interfere with men’s ability to make accurate judgments and to be in control acted as a deterrent to excessive alcohol consumption, or drinking at all, before sexual encounters. At the same time, men saw ‘alcohol as facilitating contact-seeking’ since it could be used to manage social anxiety:

“I’m a very shy person so I must use alcohol first and then look for a sexual partner to sleep with. I can’t just open up to someone that I’m not sure if he likes having sex with men because if I open up to a normal man he will embarrass me.” (Interview 8)

The Swahili phrase “mwanaume wa kawaida” was used to describe a man who is not a homosexual person. Its meaning, i.e. normal man, implies that participants might have internalised aspects of the stigma around homosexuality. Stigma was a heavy burden for participants who mentioned ‘using alcohol and drugs as coping strategies’ to handle the resulting pressure from discriminatory attitudes and behaviours:

“I think stigma is the key because it leads to stress and loneliness so it pushes us to turn to alcohol and drug abuse. You know there is a bad feeling caused by stigma from family and friends so alcohol helps in forgetting it for a while.” (Interview 1)

‘Lacking trust in partners creating a norm of using condoms’ emerged as a category. Men discussed the problems associated with not having a complete picture of partners’ sexual life:

“I only rely on the use of protection. It doesn’t matter how much I trust a person, I must use protection to avoid infections. Most of us have so many partners and I can never know if he [my partner] has others.” (Interview 1)

The perception that MSM had multiple concurrent partners helped to promote consistent condom use but it also inhibited participants’ ability to trust their partners. At the same time, when reflecting on their intimate lives, men described that ‘being involved emotionally justified condomless sex’. Feelings towards intimate partners appeared to explain why some men engaged in sex without condom: “With one of my partners I don’t use protection and this is because I love him.” (Interview 5). Being in love was a typical reason for condomless sex and closely related to trust, which appeared to contribute to the attitude that condom use therefore was redundant: “I trust him [my partner] so why should we use a condom? We love each other.” (Interview 2).

‘Being abandoned if insisting on condom’ with intimate partners was seen as a possible outcome, and rendered consistent condom use more difficult:

“Using protection is a personal decision. But the reality is that some men may insist to have unprotected sex, and if we love them, we may end up not using protection for the fear of being dumped.” (Interview 3)

The inability to negotiate for condom use was influenced by the belief that agreeing to condomless sex was the only way to sustain the relationship in an environment where it was difficult to find new partners.

Participants stressed ‘being pushed into sex work due to discriminatory attitudes’, which acted as a barrier to getting a regular employment and an income:

“We need to get jobs because many people don’t like to employ us. This causes many MSM to work as commercial sex workers.” (Interview 10)

Being involved in sex work had different implications and meanings. It could include survival sex, when participants were left with limited options to gain an income:

“When I was 17 I was kicked out from my house after my father found out that I had a boyfriend. I stayed with a friend for a while but needed money, and he linked me up with a man that was ready to pay for sex. That’s how I ended up selling sex. So I can get money to get by.” (Interview 9).

However, the financial aspects related to selling sex were not always mentioned explicitly. When describing their intimate lives, some partners were referred to as clients. A distinction was made between “commercial partners” and “permanent partners”, and intimacy between participants and clients contributed to ‘lowering incentives for condom use’:

“I try to use condoms with my clients. But it depends. Really. You see, I have seven boyfriends. Most of them are commercial partners and one is my permanent partner. We don’t use condom. The others, it depends. Some of them are really close to me and we know each other. So we don’t always need to use protection.” (Interview 1)

The quotation suggests that participants’ perceived level of intimacy and familiarity with any given partner was a decisive factor with regard to whether condoms were seen as necessary or not.

### Needing social support but fearing consequences due to exposure

Social networks provided critical avenues for information and emotional support. Being able to confide in trusted individuals and discuss partners and sex life contributed to increased insights about sexual risk-taking. Yet, using concealment as a strategy to remain unexposed as MSM caused anxiety about exposure to family in the context of existing cultural taboos around homosexuality. Men such as “Julius”, a self-identified gay man in his early twenties, made clear divisions between their family and social lives. Julius described his efforts to keep his gay friends secret from his family. While his friends remained as an important source of support and assistance, he did not dare to be seen with peers at places where family members could be present. Julius feared that family members would make assumptions about his sexual orientation if they saw him together with men who appeared feminine.

As it emerged from the analysis, ‘friends advising on sexual life’ were considered as important for own health and safety, as the information exchange between peers was considered helpful for decision-making:

“I love my friends and they have a special position in my life. They can advise me on issues of having a single partner, practicing protected sex and so many things that can keep me from getting HIV and other STIs.” (Interview 5)

The possibility of remaining non-disclosed as MSM in Tanga was discussed and the ‘small city was compromising privacy’:

“Most gays don’t know how to keep the secret, they like showing off to everyone that they are sweet. But it’s small here and people like to talk. If you want to avoid stigma then you have to behave like a straight man all the time. Otherwise people will recognise you.” (Interview 6)

Here, the word “sweet” referred to someone with feminine attributes and appearance. Participants who defined themselves as “tops” (i.e. preferring the insertive role during anal intercourse) believed that “bottoms” (i.e. preferring the receptive role) were more feminine, and hence less disposed towards concealing behaviours that could attract people’s attention.

Men expressed mixed sentiments about the use of social media with some being insecure about how to use Internet and others noting its considerable advantage in keeping contact with friends and lovers:

“These networks have a great influence because I can meet people in Facebook and create a group of friends where we share a lot of information and make new friends. Sometimes I can get sexual partners.” (Interview 9)

While Internet was used for different purposes, MSM generally agreed on its potential as a medium to connect with other men. ‘Family filling an important function for well-being’ was mentioned repeatedly throughout the interviews. Despite the often negative attitudes participants met in regard to own sexuality, siblings and relatives were critical catalysts for well-being:

“My sisters are so important in my life and they advise me on various issues. They always tell me to study hard when I’m down.” (Interview 5)

Yet, connections between the pervasive discourse around homosexuality and the family’s reaction were discussed, and ‘fearing consequences of exposure to family’ constituted an integral element:

“Being gay puts the family to great shame so I think they may even kick me out, and I may even be stigmatised by the whole society. Sometimes I hide when I meet people who know me because of the fear that the news can reach my family.” (Interview 10)

Since being gay was related to social and cultural taboos and despised images, MSM believed that having their sexual orientation disclosed to family members would result in bringing shame on the rest of the family, thus risking rejection and social exclusion.

### Challenged by stigma in healthcare settings

Perceived as well as enacted stigma prevented timely access to healthcare services. Suspicious fellow patients and insensitive healthcare workers rendered it difficult to use available services and resulted in men resorting to self-medication as a safer alternative for treatment. This was closely related to participants’ sexual positioning. “Bottoms” (“anayetiwa” in Swahili) were believed to fear healthcare workers to a greater extent than “tops” (“anayeingilia”) since the former category experienced more problems in concealing their sexual practices during interaction with the doctor or nurse. “Bottom men” were therefore easier targets for discriminatory practices by healthcare workers.

Men perceived the healthcare climate as inimical to their health and broader ‘societal attitudes against homosexuality spilling over to treatment’ of MSM clients were believed to be the main explanation: “You know homosexuality is not culturally acceptable so if a doctor finds out that you are a bottom he will stigmatise you.” (Interview 2). Participants experienced homosexuality as a behaviour or lifestyle that was socially disapproved of, and consequently, men felt a general sense of vulnerability because of unaddressed healthcare needs.

Accounts revealed how healthcare workers were ‘distinguishing between participants and other patients’. MSM described that they felt ignored and were left “for hours without getting any attention” (Interview 7), whereby they eventually gave up and returned home, resulting in lack of treatment. ‘Being denied services’ was a recurring topic. One man described the abuse by a medical doctor when she realised that he engaged in sex with other men:

“The doctor told me to use a condom every time I have sex. I told her that I have a boyfriend and I wanted to know if I could get HIV if I’m having sex with other men? She was so shocked and she didn’t believe that I was gay. She asked me if I’m crazy and told me that she couldn’t answer my questions because I’m not in a relationship with a woman. She was so shocked that she kicked me out of her room.” (Interview 9)

‘Worrying about exposure of sexual orientation’ was a problem for participants. MSM described their concerns as hindering their ability to seek healthcare assistance: “It affects me so much because I can’t go to the hospital whenever I get sick.” (Interview 8). Instead, some participants chose to endure their pain and avoided hospitals and health centres altogether. ‘Intimidatingly large patient volumes at public hospitals’ were also described as a problem, as it potentially could expose men to stigmatising situations. Participants experienced these crowds as stressful and felt intrusively monitored by those waiting and believed other patients were gossiping about them. ‘Opting for private hospitals’ when possessing the financial means was a preferred strategy due to their smaller size:

“When I have money I go to private hospitals where most patients are busy reading newspapers and they don’t have time to look at other patients. There is a huge problem in public hospitals because there is a lot of poor people who like gossiping.” (Interview 3)

In contrast to public hospitals, MSM appeared to feel comfortable at private hospitals due to the higher level of discretion and privacy at these places. ‘Perceived stigma resulting in harmful sexual behaviours’ was described as a direct consequence of the lack of possibilities to consult healthcare workers:

“Sometimes we can keep having sex with other men even when we have anal sores and abrasions and end up putting our health and our partner’s health in great danger. But we can’t go to the hospital and open up to the doctor.” (Interview 9)

‘Knowing a doctor’ was a considerable advantage as one could “pay quickly for services” (Interview 1). Using personal contacts removed some of the barriers related to healthcare-seeking since MSM already a priori knew they would receive assistance against payment. ‘Resorting to self-medication’ constituted another preferred strategy. Participants were ‘receiving advice from social networks’ regarding what drugs to use for their symptoms:

“We have a network of friends, so we call and ask each other about treatment of various diseases. Then I just go and buy it [the drug] at the drugstore.” (Interview 8)

When reflecting on these networks MSM described how their peers helped them to diagnose certain symptoms and shared information about what medication to obtain without having to seek assistance first from formal healthcare services.

## Discussion

These data are among the first to explore sexual behaviours and healthcare-seeking in relation to perceived risks and constraints among MSM in a provincial Tanzanian city. Participants reflected on HIV/STI risks, sexual relationships, and healthcare experiences and described how their social environment either restricted them or supported them. ‘Acting within an increasingly confined space’, the overarching theme, captures the almost omnipotent role of stigma in men’s lives. Perceived stigma created barriers for sexual and social networks and healthcare-seeking, and discriminating attitudes within the labour sector forced men into sex work to survive. Despite coping strategies, men were limited in their choices of partners, and the fear of rejection and stigma from the partner influenced their exposure to risky sexual scenarios. All in all, stigma seemed to have a considerable impact on their health and well-being. Thus, stigma may ultimately be a major impediment to attaining the global target of ending the AIDS epidemic by 2030 [33].

### Intimacy and risk perception

These findings are similar to other studies from the metropolitan city of Dar es Salaam, Tanzania, where MSM reported high levels of alcohol abuse, commercial sex work, and stigma in healthcare [1, 5, 6]. In our study, men expected partners to be involved in multiple concurrent partnerships, and also they themselves engaged in simultaneous sexual relations. This raises concerns about potential exposure to HIV/STI infection through own and partners’ sexual networks [34]. While men cited lack of trust as a reason for using condom, the interviews revealed that intimacy attenuated risk perceptions and reduced incentives to use condoms with some partners. The latter might give additional in-depth insight into the high rates of condomless sex that have been reported previously in Tanzania [6]. Intimacy as a risk factor in same-sex relationships has previously been assessed, and qualitative studies have found that the condomless act itself represents a level of intimacy, which is valued higher than the risk of infection [35, 36]. In addition, the findings regarding commercial sex work indicated that intimacy influenced risk perceptions regarding sexual partners. Although men employed protective strategies with strict condom use in commercial sex work, the interviews indicated that exemptions were made for certain clients, as the distinction between commercial and intimate partners sometimes could become diffuse. Previous research has suggested that such diffuseness may influence own risk perception and motivation to use condoms in sexual relationships. Okal and colleagues found in their study of MSM sex workers in Mombasa, Kenya, that condom use seemed to decrease as MSM formed closer ties with clients [37]. In light of the seemingly existing concurrent sexual relations that men in our study engaged in, it is critical to address level of emotional involvement as a risk factor for HIV infection both in non-commercial and commercial sexual relations.

Homosexual behaviours are illegal in Tanzania, and there are few HIV prevention and treatment programmes explicitly targeting MSM [27]. Our data suggest, however, that there is a need to bolster MSM’s motivation to maintain consistent condom use with partners and enhance condom negotiation skills. MSM could benefit from interventions that address their self-efficacy in using condoms and influencing condom use, which also would dispel common myths about intuition as a reliable source of determining risk of infection. Previous studies from elsewhere in Africa have demonstrated that MSM specific programmes may have a positive effect on condom use with partners [38–40]. However, the recent suspension of MSM outreach programmes in Tanzania poses a major obstacle for those efforts–in particular those that are driven by the LGBT community [41, 42]. Given the high prevalence of HIV in the MSM community this is a serious concern. Successful skills training methods to promote condom use need to be incorporated into on-going outreach programmes that already have gained legitimacy and trust among the MSM population. The government’s ban on MSM outreach programmes risks creating a gap in the efforts to reach marginalised populations, hence increasing their vulnerability to HIV infection, which would undermine the country’s endeavours to advance HIV prevention.

### Barriers regarding condom use

Study participants cited partners’ resistance towards using condoms, and they felt forced to engage in condomless sex in order to avoid rejection. One might speculate that rejection has more severe consequences in a setting where fewer MSM partners are available and options to engage sexually and/or commit emotionally are more limited. This raises the need to address concerns within the MSM population itself regarding the use of condoms. In a study on 86 Ugandan MSM, Musinguzi et al. found a number of reasons why participants engaged in sex without condoms [43]. Reasons for engaging in condomless sex cited by MSM in their study included, in addition to concerns about comfort and quality of condoms also beliefs that same-sex activity was safe. Efforts to increase sexual health knowledge are required also in the Tanzanian context, and further studies need to identify the barriers that exist for condom use in order to inform targeted messages about condom and lubricant preparedness. However, since 2016, the Tanzanian government has prohibited the use of lubricants as an HIV intervention [41]. Condoms and water- or silica-based lubricants are highly effective in preventing STIs, including HIV [44]. The government should carefully consider the current ban in light of the most recent scientific knowledge and evaluate how the ban might influence not only the HIV epidemic in key populations but also in the general population, as many MSM also have female partners [6].

### Discrimination and stigma in healthcare services

This study showed that MSM in a provincial city experienced unmet needs with regard to healthcare services due to discrimination and stigma, and thus the findings are similar to what has been revealed in larger cities in Sub-Saharan Africa [10, 14–16]. These findings are also well in line with those from the larger quantitative study that this study was nested within, where 67.7% of the respondents perceived that doctors and nurses stigmatised gay men [26]. Our findings revealed that insensitive providers and fear of being stigmatised by patients constituted barriers to healthcare and pushed men into self-medication. Healthcare workers need to be trained to deliver competent services that will encourage MSM to seek healthcare. In neighbouring Kenya, MSM-sensitive online training of healthcare workers has reduced homophobic attitudes [45]. Given that the Tanzanian government, in its suspension of MSM drop-in centres, places the responsibility of MSM’s HIV testing and treatment on regular health centres, de-stigmatising interventions in these settings are urgently needed, if at all feasible [41].

### The role of social media

As noted in previous research from rural South Africa, men in this study used social media to communicate with each other–although to a varying extent [23]. Recent threats by the government to use social media to track down suspected gay persons will presumably have an influence upon how MSM use such media [46]. MSM will need to be careful about how they socialise online and with whom. Yet, social and online media have the potential to reach MSM with sexual health information–in particular in the context of a smaller city where formal MSM specific initiatives may be limited [47]. HIV prevention strategies that are technology-based have shown promising results in reducing condomless sex among MSM in other contexts [48–52]. By equipping Tanzanian MSM with HIV/STI knowledge, dispelling condom myths and sharing real life stories, web-based interventions could contribute towards national HIV targets. Advertisements on gay websites, for example, could be an entry point to reach MSM with sexual health messages while still maintaining their privacy. Such advertisements could then redirect those who are interested to web-based prevention programmes with tailored information and resources. However, it will be important that these providers maintain a strict privacy preservation policy with technically sound solutions that protect their users from data hacking [53].

### Psychosocial well-being and sexual risk-taking

Being MSM in a provincial city may have created additional fears of being exposed as gay and hence subjected to stigmatisation. Men cited the small setting as a barrier to their possibilities for remaining anonymous as MSM. The extent to which this affects the mental health of MSM and the potential effect of poor mental health on sexual risk-taking are yet to be explored in this setting. Previous research from other settings has shown a relationship between poor mental health and sexual-risk taking among MSM in environments characterised by intolerance [54, 55]. Preston et al. in their study of rural MSM in the US suggested that MSM resorted to sexual risk-taking as a way of coping with the stress caused by their constant efforts to avoid disclosure and thus the risk of becoming stigmatised [55]. Such stress would probably be of greater concern in a smaller setting where there are fewer possibilities to remain anonymous as MSM and emphasises the need for interventions that also address psychosocial well-being. In Kenya, Secor et al. found a relationship between sexual stigma and depression in a study among 112 MSM in the town of Mtwapa [56]. The need for targeted mental healthcare for MSM populations has been established in a large body of literature [5, 57–60]. However, given that Tanzania is a low-income country and that homosexuality is illegal, it is questionable to what extent specialised mental healthcare services for this population could be implemented. Instead, MSM could benefit from the establishment of support groups, similar to those that exist for people living with HIV [61]. Participants in our study had access to a social network of other MSM, and peer-led initiatives could levy on these networks to build capacity in leadership and communication skills among selected peers that can be used to establish and maintain support groups.

### Methodological considerations

The study has several strengths. It is one of the first studies in sub-Saharan Africa to explore sexual behaviour and healthcare needs among MSM residing in areas outside mainstream gay venues. Prior to the study, the data collector (JS) spent considerable time in Tanga to gain an understanding of the specific situation of MSM in this setting [62]. During the process of analysis, each step was documented to assure dependability and confirmability, and detailed quotations were provided to illuminate the interpretation of the findings [62]. However, several limitations need to be acknowledged. First, as this was a nested study within a larger quantitative study that used respondent-driven sampling (RDS), every 10^th^ participant was recruited for the interview study to minimise potential recruitment bias. A more purposive sampling strategy, which targeted men with specific characteristics, such as men with positive and negative experiences from the healthcare sector, could have yielded other perspectives [63, 64]. Nevertheless, after reviewing the transcripts it was agreed that saturation had been achieved in the collected data concerning risk perceptions, sexual practices, and healthcare needs.

Furthermore, most participants were part of a MSM network, presumably due to the RDS recruitment strategy that utilises participants’ own networks to generate study subjects. While we initially selected seeds with different socio-demographic characteristics to ensure a wider representation, MSM who were not members of a MSM social network were excluded. Future studies should also include MSM without MSM social networks, as their experiences with regard to risk and healthcare might differ. It is not unlikely that such persons might be at even more increased risk of HIV/STI infection due to the lack of informative and emotional support that MSM social networks offer. Finally, since participants’ original language was translated into English there is a risk that certain words or expressions were changed unintentionally by the translator. However, to assess the accuracy of the translation, one of the co-authors, who is a native speaker in Swahili, cross-checked a number of the transcripts.

## Conclusions

The findings of this qualitative study suggest that sexual stigma is a main driver of sexual risk practices among MSM in a provincial city and prevents them from timely healthcare access. The current climate around homosexuality in Tanzania requires careful consideration of the practicality of any intervention that intends to address the MSM population. Creating safe cyber networks that provide accurate and targeted HIV/AIDS and STI information might provide an opportunity to reach this population. Specifically designed messages concerning the sexual health of MSM could be displayed in these networks to confer knowledge and support individuals in their choices. Such initiatives might be even more urgent in smaller towns and rural areas where MSM specific programmes are more limited than in urban areas.

## Supporting information

S1 Text Thematic interview guide- English. (DOCX)

S2 Text Thematic interview guide- Swahili. (DOCX)

## References

[1] Ross MW, Nyoni J, Ahaneku HO, Mbwambo J, McClelland RS, McCurdy SA. High HIV seroprevalence, rectal STIs and risky sexual behaviour in men who have sex with men in Dar es Salaam and Tanga, Tanzania. BMJ Open. 2014;4(8): e006175 doi: 10.1136/bmjopen-2014-006175 25168042 10.1136/bmjopen-2014-006175 PMC4156794

[2] Leshabari MT, Mmbaga EJ, Mpembeni R, Moen K. Prevalence of the Human Immunodeficiency Virus, other sexually transmitted infections, and health-related perceptions, reflections, experiences and practices among men having sex with men in Dar es Salaam. 2013. Available from: http://tacaids.go.tz/tacaids/images/sampledata/documents/MSM-report-FINAL.pdf. Cited 02 March 2017.

[3] Larsson M, Agardh A, Ross MW, Månsson SA, Nyoni J, Shio J. Being Forced to Become Your Own Doctor: Men Who Have Sex with Men's Experiences of Stigma in the Tanzanian Healthcare System. Int J Sex Health. 2016;28(2):163–75. doi: 10.1080/19317611.2016.1158763 28491204 10.1080/19317611.2016.1158763 PMC5421638

[4] Magesa DJ, Mtui LJ, Abdul M, Kayange A, Chiduo R, Leshabari MT, et al Barriers to men who have sex with men attending HIV related health services in Dar es Salaam, Tanzania. Tanzan J Health Res. 2014;16(2):118–26. 26875306 10.4314/thrb.v16i2.8

[5] Ahaneku H, Ross MW, Nyoni JE, Selwyn B, Troisi C, Mbwambo J, et al Depression and HIV risk among men who have sex with men in Tanzania. AIDS Care. 2016;28(sup1):140–7. 27002772 10.1080/09540121.2016.1146207 PMC4859320

[6] Bui TC, Nyoni JE, Ross MW, Mbwambo J, Markham CM, McCurdy SA. Sexual motivation, sexual transactions and sexual risk behaviors in men who have sex with men in Dar es Salaam, Tanzania. AIDS Behav. 2014;18(12):2432–41. doi: 10.1007/s10461-014-0808-x 24890184 10.1007/s10461-014-0808-x PMC4229400

[7] Nyoni JE, Ross MW. Condom use and HIV-related behaviors in urban Tanzanian men who have sex with men: a study of beliefs, HIV knowledge sources, partner interactions and risk behaviors. AIDS care. 2013;25(2):223–9. doi: 10.1080/09540121.2012.699671 22788911 10.1080/09540121.2012.699671

[8] Ross MW, Larsson M, Nyoni JE, Agardh A. Prevalence of STI symptoms and high levels of stigma in STI healthcare among men who have sex with men in Dar es Salaam, Tanzania: a respondent-driven sampling study. Int J STD AIDS. 2016 12 2. [Epub ahead of print]. 10.1177/0956462416683625 27913795

[9] Risher K, Adams D, Sithole B, Ketende S, Kennedy C, Mnisi Z, et al Sexual stigma and discrimination as barriers to seeking appropriate healthcare among men who have sex with men in Swaziland. J Int AIDS Soc. 2013;16(3 Suppl 2):18715. 24242263 10.7448/IAS.16.3.18715 PMC3833105

[10] Lane T, Mogale T, Struthers H, McIntyre J, Kegeles SM. “They see you as a different thing”: the experiences of men who have sex with men with healthcare workers in South African township communities. Sex Transm Infect. 2008; 84(6):430–3. doi: 10.1136/sti.2008.031567 19028941 10.1136/sti.2008.031567 PMC2780345

[11] Cheikh Ibrahima N, Placide T, Weiss E, Moustapha D, Youssoupha N, Amadou Mody M, et al 'It's Raining Stones': Stigma, Violence and HIV Vulnerability among Men Who Have Sex with Men in Dakar, Senegal. Cult Health Sex. 2003;5(6):499–512.

[12] Baral S, Adams D, Lebona J, Kaibe B, Letsie P, Tshehlo R, et al A cross-sectional assessment of population demographics, HIV risks and human rights contexts among men who have sex with men in Lesotho. J Int AIDS Soc. 2011;14(36). 10.1186/1758-2652-14-36 PMC3146892 21726457

[13] Adebajo SB, Eluwa GI, Allman D, Myers T, Ahonsi BA. Prevalence of internalized homophobia and HIV associated risks among men who have sex with men in Nigeria. Afr J Reprod Health. 2012;16(4):21–8. 23444540

[14] Fay H, Baral SD, Trapence G, Motimedi F, Umar E, Iipinge S, et al Stigma, health care access, and HIV knowledge among men who have sex with men in Malawi, Namibia, and Botswana. AIDS Behav. 2011;15(6):1088–97. doi: 10.1007/s10461-010-9861-2 21153432 10.1007/s10461-010-9861-2

[15] Wanyenze RK, Musinguzi G, Matovu JK, Kiguli J, Nuwaha F, Mujisha G, et al “If You Tell People That You Had Sex with a Fellow Man, It Is Hard to Be Helped and Treated”: Barriers and Opportunities for Increasing Access to HIV Services among Men Who Have Sex with Men in Uganda. PloS One. 2016;11(1):e0147714 doi: 10.1371/journal.pone.0147714 26808653 10.1371/journal.pone.0147714 PMC4726486

[16] Wirtz AL, Kamba D, Jumbe V, Trapence G, Gubin R, Umar E, et al A qualitative assessment of health seeking practices among and provision practices for men who have sex with men in Malawi. BMC Int Health Hum Rights. 2014;14(20). 10.1186/1472-698X-14-20 PMC4049421 24893654

[17] Imrie J, Hoddinott G, Fuller S, Oliver S, Newell. Why MSM in Rural South African Communities Should be an HIV Prevention Research Priority. AIDS Behav. 2013;17(Suppl 1):70–6. 10.1007/s10461-012-0356-1 PMC3627851 23196857

[18] Wade AS, Kane CT, Diallo PAN, Diop AK, Gueye K, Mboup S, et al HIV infection and sexually transmitted infections among men who have sex with men in Senegal. AIDS. 2005;19(18):2133–40. 16284463 10.1097/01.aids.0000194128.97640.07

[19] Sanders EJ, Graham SM, Okuku HS, van der Elst EM, Muhaari A, Davies A, et al HIV-1 infection in high risk men who have sex with men in Mombasa, Kenya. AIDS. 2007;21(18):2513–20. doi: 10.1097/QAD.0b013e3282f2704a 18025888 10.1097/QAD.0b013e3282f2704a

[20] Smith AD, Tapsoba P, Peshu N, Sanders EJ, Jaffe HW. Men who have sex with men and HIV/AIDS in sub-Saharan Africa. Lancet. 2009;374(9687):416–22. doi: 10.1016/S0140-6736(09)61118-1 19616840 10.1016/S0140-6736(09)61118-1

[21] Muraguri N, Temmerman M, Geibel S. A decade of research involving men who have sex with men in sub-Saharan Africa: current knowledge and future directions. SAHARA J. 2012;9(3):137–47. doi: 10.1080/17290376.2012.744176 23237068 10.1080/17290376.2012.744176

[22] Tanzania Ministry of Health and Social Welfare, National AIDS Control Programme. Consensus Estimates on Key Population Size and HIV Prevalence in Tanzania. 2014. Available from: https://www.healthpolicyproject.com/pubs/391_FORMATTEDTanzaniaKPconsensusmtgreport.pdf. Cited 10 March 2017.

[23] Maleke K, Makhakhe N, Peters RP, Jobson G, De Swardt G, Daniels J, et al HIV risk and prevention among men who have sex with men in rural South Africa. Afr J AIDS Res. 2017;16(1):31–8. doi: 10.2989/16085906.2017.1292925 28367747 10.2989/16085906.2017.1292925

[24] National Bureau of Statistics, Ministry of Finance Dar es Salaam, Office of Chief Government Statistician President’s Office, President's Office of Finance Economy and Development Planning Zanizbar. 2012 Population and Houseing Census. Population Distribution by Administrative Areas. 2013. Available from: http://www.tzdpg.or.tz/fileadmin/documents/dpg_internal/dpg_working_groups_clusters/cluster_2/water/WSDP/Background_information/2012_Census_General_Report.pdf. Cited 02 January 2017.

[25] Sharf N. Stronger voices for LGBT rights in Tanzania- A summary. Copenhagen: LGBT Danmark–The Danish National Organisation for Gay Men, Lesbians, Bisexuals and Transgender Persons 2014 Available from: http://lgbt.dk/wp-content/uploads/Stronger-Voices-Summary-Final.pdf. Cited 15 May 2017.

[26] Ross MW, Larsson M, Jacobson J, Nyoni J, Agardh A. Social networks of men who have sex with men and their implications for HIV/STI interventions: results from a cross-sectional study using respondent-driven sampling in a large and a small city in Tanzania. BMJ Open. 2016;6(11): e012072 doi: 10.1136/bmjopen-2016-012072 27864245 10.1136/bmjopen-2016-012072 PMC5129084

[27] Government of Tanzania. The sexual offences special provisions Act, 1998. 1998. Avaialble from: http://www.lrct.go.tz/?wpfb_dl=170. Cited 10 January 2017.

[28] Odoyo R. The other Tanzanians. Landscape analysis of the human rights of Sex Workers & LGBTI communities in Tanzania 2015–2016. 2015 Nairobi: UHAI EASHRI Available from: http://globalphilanthropyproject.org/wp-content/uploads/2016/03/Tanzania-Baseline_ENG_print.pdf. Cited 15 May 2017.

[29] Kvale S, Brinkmann S. InterViews: learning the craft of qualitative research interviewing 2nd ed. Los Angeles: Sage Publications; 2009.

[30] Graneheim UH, Lundman B. Qualitative content analysis in nursing research: concepts, procedures and measures to achieve trustworthiness. Nurse Educ Today. 2004; 24(2):105–12. doi: 10.1016/j.nedt.2003.10.001 14769454 10.1016/j.nedt.2003.10.001

[31] Krippendorff K. Content analysis: An introduction to its methodology 2nd ed. Thousand Oaks: Sage Publications, Inc.; 2004.

[32] Dey I. Qualitative data analysis: a user-friendly guide for social scientists London: Routledge; 1993.

[33] Sidibé M, Loures L, Samb B. The UNAIDS 90–90–90 target: a clear choice for ending AIDS and for sustainable health and development. J Int AIDS Soc. 2016;19(1): 21133 doi: 10.7448/IAS.19.1.21133 27424601 10.7448/IAS.19.1.21133 PMC4947868

[34] Van Tieu H, Nandi V, Frye V, Stewart K, Oquendo H, Bush B, et al Concurrent Partnerships and HIV Risk among Men Who Have Sex with Men in New York City. Sex Transm Dis. 2014;41(3):200–8. doi: 10.1097/OLQ.0000000000000090 24521727 10.1097/OLQ.0000000000000090 PMC4171743

[35] Carballo-Dieguez A, Dolezal C. HIV risk behaviors and obstacles to condom use among Puerto Rican men in New York City who have sex with men. Am J Public Health. 1996;86(11):1619–22. 8916531 10.2105/ajph.86.11.1619 PMC1380700

[36] McLean J, Boulton M, Brookes M, Lakhani D, Fitzpatrick R, Dawson J, et al Regular partners and risky behaviour: why do gay men have unprotected intercourse? AIDS Care. 1994;6(3):331–41. doi: 10.1080/09540129408258645 7948089 10.1080/09540129408258645

[37] Okal J, Luchters S, Geibel S, Chersich MF, Lango D, Temmerman M. Social context, sexual risk perceptions and stigma: HIV vulnerability among male sex workers in Mombasa, Kenya. Cult Health Sex. 2009;11(8):811–26. doi: 10.1080/13691050902906488 19484638 10.1080/13691050902906488

[38] Larmarange J, Wade AS, Diop AK, Diop O, Gueye K, Marra A, et al Men Who Have Sex with Men (MSM) and Factors Associated with Not Using a Condom at Last Sexual Intercourse with a Man and with a Woman in Senegal. PloS One. 2010;5(10): e13189 doi: 10.1371/journal.pone.0013189 20957157 10.1371/journal.pone.0013189 PMC2950158

[39] Henry E, Marcellin F, Yomb Y, Fugon L, Nemande S, Gueboguo C, et al Factors associated with unprotected anal intercourse among men who have sex with men in Douala, Cameroon. Sex Transm Infect. 2010;86(2):136–40. doi: 10.1136/sti.2009.036939 19703845 10.1136/sti.2009.036939

[40] Geibel S, Luchters S, King'Ola N, Esu-Williams E, Rinyiru A, Tun W. Factors associated with self-reported unprotected anal sex among male sex workers in Mombasa, Kenya. Sex Transm Dis. 2008;35(8):746–52. doi: 10.1097/OLQ.0b013e318170589d 18650772 10.1097/OLQ.0b013e318170589d

[41] Ministry of Health, Community Development, Gender, Elderly and Children. Statement by the Minister for Health, Community Development, Gender, Elderly and Children, Hon. Ummy Mwalimu on key populations HIV services in Tanzania: 27th October, 2016. Dar es Salaam: Ministry of Health, Community Development, Gender, Elderly and Children. 2016. Avaialble from: https://www.facebook.com/afyatz/posts/1230922863594845. Cited 11 May 2017.

[42] Sieff K. Tanzania suspends U.S.-funded AIDS programs in a new crackdown on gays. The Washington Post. 23 November 2016. Avaialble from: https://www.washingtonpost.com/world/africa/tanzania-suspends-us-funded-aids-programs-in-a-new-crackdown-on-gays/2016/11/23/ec6ced6e-ab5c-11e6-8f19-21a1c65d2043_story.html?utm_term=.c3896fa07d77. Cited 11 May 2017.

[43] Musinguzi G, Bastiaens H, Matovu JK, Nuwaha F, Mujisha G, Kiguli J, et al Barriers to Condom Use among High Risk Men Who Have Sex with Men in Uganda: A Qualitative Study. PloS One. 2015;10(7): e0132297 doi: 10.1371/journal.pone.0132297 26172374 10.1371/journal.pone.0132297 PMC4501754

[44] Joint United Nations Programme on HIV/AIDS. Condom and lubricant programming in high HIV prevalence countries 2014 Geneva: UNAIDS Avaialble from: http://www.unaids.org/sites/default/files/media_asset/condoms_guidancenote_en.pdf. Cited 11 5 2017.

[45] van der Elst EM, Smith AD, Gichuru E, Wahome E, Musyoki H, Muraguri N, et al Men who have sex with men sensitivity training reduces homoprejudice and increases knowledge among Kenyan healthcare providers in coastal Kenya. J Int AIDS Soc. 2013;16(4 Suppl 3): 18748. 24321111 10.7448/IAS.16.4.18748 PMC3852129

[46] Makoye K. Gay sex is illegal in Tanzania and punishable by up to 30 years in prison Thomson Reuters Foundation News 8 9 2016 Avaialble from: http://news.trust.org/item/20160908164618-ch8u3/. Cited 13 May 2017.

[47] Williams ML, Bowen AM, Horvath KJ. The Social/Sexual Environment of Gay Men Residing in a Rural Frontier State: Implications for the Development of HIV Prevention Programs. J Rural Health. 2005;21(1):48–55. 15667009 10.1111/j.1748-0361.2005.tb00061.x PMC2614671

[48] Chiasson MA, Shaw FS, Humberstone M, Hirshfield S, Hartel D. Increased HIV disclosure three months after an online video intervention for men who have sex with men (MSM). AIDS Care. 2009;21(9):1081–9. doi: 10.1080/09540120902730013 20024766 10.1080/09540120902730013

[49] Bauermeister JA, Pingel ES, Jadwin-Cakmak L, Harper GW, Horvath K, Weiss G, et al Acceptability and Preliminary Efficacy of a Tailored Online HIV/STI Testing Intervention for Young Men who have Sex with Men: The Get Connected! Program. AIDS Behav. 2015;19(10):1860–74. doi: 10.1007/s10461-015-1009-y 25638038 10.1007/s10461-015-1009-y PMC4522230

[50] Brennan DJ, Lachowsky NJ, Georgievski G, Rosser BR, MacLachlan D, Murray J. Online Outreach Services Among Men Who Use the Internet to Seek Sex With Other Men (MISM) in Ontario, Canada: An Online Survey. J Med Internet Res. 2015;17(12):e277 doi: 10.2196/jmir.4503 26681440 10.2196/jmir.4503 PMC4704956

[51] Bowen AM, Williams ML, Daniel CM, Clayton S. Internet based HIV prevention research targeting rural MSM: feasibility, acceptability, and preliminary efficacy. J Behav Med. 2008;31(6):463–77. doi: 10.1007/s10865-008-9171-6 18770021 10.1007/s10865-008-9171-6 PMC2614302

[52] Schnall R, Travers J, Rojas M, Carballo-Diéguez A. eHealth Interventions for HIV Prevention in High-Risk Men Who Have Sex With Men: A Systematic Review. J Med Internet Res. 2014;16(5):e134 doi: 10.2196/jmir.3393 24862459 10.2196/jmir.3393 PMC4051738

[53] Hoang NP, Asano Y, Yoshikawa M, editors. Your neighbors are my spies: Location and other privacy concerns in GLBT-focused location-based dating applications. 2016. 18th International Conference on Advanced Communication Technology (ICACT); Jan 31-Feb 03 2016.

[54] Preston DB, AR, Kassab CD, Cain RE, Schulze FW, Starks MT. The influence of stigma on the sexual risk behavior of rural men who have sex with men. AIDS Educ Prev. 2004;16(4):291–303. doi: 10.1521/aeap.16.4.291.40401 15342332 10.1521/aeap.16.4.291.40401

[55] Preston DB, D'Augelli AR, Kassab CD, Starks MT. The relationship of stigma to the sexual risk behavior of rural men who have sex with men. AIDS Educ Prev. 2007;19(3):218–30. doi: 10.1521/aeap.2007.19.3.218 17563276 10.1521/aeap.2007.19.3.218

[56] Secor AM, Wahome E, Micheni M, Rao D, Simoni JM, Sanders EJ, et al Depression, substance abuse and stigma among men who have sex with men in coastal Kenya. AIDS. 2015;29(Suppl 3):S251–9. 26562814 10.1097/QAD.0000000000000846 PMC4706380

[57] Li HH, Holroyd E, Lau J, Li X. Stigma, Subsistence, Intimacy, Face, Filial Piety, and Mental Health Problems Among Newly HIV-Diagnosed Men Who Have Sex With Men in China. J Assoc Nurses AIDS Care. 2015;26(4):454–63. doi: 10.1016/j.jana.2015.02.004 26066696 10.1016/j.jana.2015.02.004 PMC4605918

[58] Lea T, de Wit J, Reynolds R. Minority stress in lesbian, gay, and bisexual young adults in Australia: associations with psychological distress, suicidality, and substance use. Arch Sex Behav. 2014;43(8):1571–8. doi: 10.1007/s10508-014-0266-6 24573397 10.1007/s10508-014-0266-6

[59] Meyer IH. Minority stress and mental health in gay men. J Health Soc Behav. 1995;36(1):38–56. 7738327

[60] Tucker A, Liht J, de Swardt G, Jobson G, Rebe K, McIntyre J, et al An exploration into the role of depression and self-efficacy on township men who have sex with men's ability to engage in safer sexual practices. AIDS Care. 2013;25(10):1227–35. doi: 10.1080/09540121.2013.764383 23387517 10.1080/09540121.2013.764383

[61] Cloete A, Simbayi LC, Kalichman SC, Strebel A, Henda N. Stigma and discrimination experiences of HIV-positive men who have sex with men in Cape Town, South Africa. AIDS Care. 2008;20(9):1105–10. doi: 10.1080/09540120701842720 18608067 10.1080/09540120701842720 PMC3320098

[62] Lincoln YS, Guba EG. Naturalistic inquiry: Beverly Hills: Sage 1985.

[63] Ravenell JE, Johnson WE Jr., Whitaker EE. African-American men's perceptions of health: a focus group study. J Natl Med Assoc. 2006;98(4):544–50. 16623067 PMC2569257

[64] Ravenell JE, Whitaker EE, Johnson WE. According to Him: Barriers to Healthcare among African-American Men. J Natl Med Assoc. 2008;100(10):1153–60. 18942276 10.1016/s0027-9684(15)31479-6

