# Supplementary material for: Acting within an increasingly confined space: A qualitative study of sexual behaviours and healthcare needs among men who have sex with men in a provincial Tanzanian city
Source: PLoS One. 2017 Aug 17;12(8):e0183265. doi: 10.1371/journal.pone.0183265 (PMC5560662; doi:10.1371/journal.pone.0183265)
Supplement: S1 Text — (DOCX) [file pone.0183265.s001.docx]

**S1 Thematic Interview Guide- English**

**Consent form- read aloud**

My name is ____. We are currently conducting a study on the perceptions and experiences of men who have sex with men here in Tanga with regards to relationships, sexual practices, social and family networks, and healthcare-seeking behaviors. The findings of the study will contribute to an improved understanding of the health needs of this population and also provide important information on how to more effectively access and engage men who have sex with men in healthcare provision and HIV/STI programming.

The study is co-coordinated by the University of Dar es Salaam and the University of Texas, US.

Your participation in the study is completely voluntary, you will not be forced to answer questions that you do not want to answer and you may end the interview at any time you want to. We would also like to assure you that all information collected in the course of the study will remain confidential. Your name will not be written on the interview scripts in a manner that someone can identify the source of information. As you will be anonymous, there are no risks associated with participation in this study.

However, this information may be used in scientific publications or presentations to increase the awareness of HIV/AIDS and STIs among men who have sex with men. Your information will be completely anonymized and no one will be able to trace your answers back to you.

The interview will take approximately one hour and I will audio record the interview. The recoridngs will be destroyed once the interview is transcribed. Should you prefer not to be recorded please let me know and I will instead take notes during the interview.

In case you have any questions, concerns or comments related to this study feel free to contact the following people who will provide you with the necessary assistance (provide card with contact information).

Do you have any questions?

**Do you agree to participate in this interview? Say “yes” or “no” and today’s date.**

**INTERVIEW QUESTIONS**

We are going to talk about various issues facing MSM here in Tanga. Please feel free and comfortable

***Sexual history***

Let us talk about your sexual history. Remember everything is confidential and you decide what you want to tell me.

When was the first time that you started having sexual feelings for men?

*Probes*

- *Do you remember what exactly happened and you learnt that you were attracted by other men?*
- *Whom were you living with during this time?*

How old were you during your first sexual intercourse?

*Probe*

- *Was it with a man or a woman?*

Please tell me more about your first male lover, where did you meet?

How old was he? Was he a little bit older than you or younger than you?

*Probe*

- How did you feel about the age difference (if a difference)

We are sometimes forced into sexual actions that we do not want to. I know this is sensitive but would you like to share with me if you have any experience of sexual coercion?

*Probes*

- *What happened? (If yes)*
- *Was it an older or younger person?*

**Current sexual life**

How is your relationship with women?

*Probes*

- *Do you have a girlfriend or several girlfriends?*
- *How long have you been together?*
- *Does she know that you are gay?*

How many girlfriends have you ever had in your life?

What sex attracts you the most?

Please tell me about your current relationship status

*Probes*

- *How many sexual partners do you have?*
- *Are they permanent or commercial partners?*
- *Where is the common place that you have been having sex at with your partner(s)?*
- *Why do you prefer these places?*

How many times do you have sex in a week?

What influences you to have sex with your partner(s)?

*Probes*

- *What role does alcohol play when you have sex?*
- *What role does drugs play when you have sex?*
- *The physical environment, for ex. a hidden corner in the street or a romantic place*

What role does the cell phone play for your sex life?

*Probes*

*- Do you use it to communicate with sex partners?*

***Social life***

Would you like to tell me about the role of your friends in life?

*Probes*

- *How do your friends influence you in making various decisions in various life aspects?*
- *With regards do your relationship with significant others, what role do your friends play? For example if you get a boyfriend, do you think that your friends may influence you in choosing the king of relationship you want to have with him?*
- *What friends could not give you advice and why?*

In your experience, what role do your friends play in helping you to find for sexual partners?

*Probe*

- *Can your friends warn you either to do something or not to do it?*

What is the influence of elders in your life?

*Probe*

- *Can elders like your parents have any influence in your life? For example, can they advice you in your love life?*

What role does your family play in your life?

*Probes*

- *Do your parents and siblings know that you have sex with other men?*
- *Why can’t you tell them? (If no)*
- *Why do you think that they accept this? (If yes)*

**Stigma**

We have been discussing about personal issues and now we are going to talk about stigma. How is the situation of stigma in Tanga?

*Probe*

- *Tell me more about it, how is it shown?*
- *How does a non-gay community view you?*

How is stigma shown at the family level?

How is stigma shown at work places?

*Probe*

- *Are you employed?*
- *What may if other workers find out that you are gay*

What if you get a problem that requires you to go to a police station? For example if you get robbed and go to report at a police station will they help you?

What role does stigma play stigma when you need to access social help whenever you need it? For example if you need to borrow money from someone?

Let us say you have seen a handsome man in the street and fall for him. How do you approach him?

*Probe*

- *Why do you use that technique?*
- *Do you think if you face him directly he will stigmatize you?*

What about stigma in public, can you share your experiences of this with me?

*Probe*

- *Have you ever been stigmatized in public just because you are a man who has sex with men?*

How does stigma make you feel especially when you or your close friends get stigmatized?

If the law could change today and allow homosexuality in the country, what do you think could change in your life?

**Stigma in healthcare**

Could you please tell me about stigma towards MSM in healthcare

*Probe*

- *How is it shown?*
- *Tell me more about how fellow patients are towards you?*
- *How are the front officers and nurses? And the doctors?*
- *Can you share with me a personal experience?*

How does stigma affect your access to diagnosis treatment of sexually transmitted diseases?

*Probe:*

- *What do you feel are the challenges in accessing healthcare services?*
- *What are the challenges that your friends face?*

What roles do you think stigma plays in relation to risky behavior like unprotected sex and drugs abuse?

What roles do you think stigma plays in relation to self-treatment, when buying medicine from the pharmacy and use it without doctor’s prescription?

**Health service analysis**

Have you ever had any symptoms of sexually transmitted diseases?

*Probe*

*- Could you please describe these symptoms? (if yes)*

When you had this problem where did you go for treatment? (If yes to above)

*Probe*

- *Why did you choose this place?*
- *Tell me more about this visit, what happened?*

Have you ever had HIV test?

*Probe*

- *Why not? (If no)*
- *Where did you test and why did you go there? (If yes)*
- *What was you experience? (If yes)*

**Sexual risk behaviors**

Let us discuss issues related to risky behavior. How do you analyze if a certain behavior is risky or not?

*Probe*

- *How do you know if a certain behavior is risky or not?*
- *How do you make choices on practices that can keep you from getting HIV and other STIs?*

Now, please tell me how do you know that your partner is in the risk of getting HIV and other sexually transmitted diseases?

*Probe*

- *For example how do you know that your partner has multiple partners, which puts you in a risk of getting HIV?*
- *What do you do if your partner doesn’t want to use protection?*

What is the impact of stigma in looking for a sexual partner?

Can you tell me how stigma affects communication with your partner?

*Probe*

*- Are there things that you can’t raise?*

Can you tell me how stigma influences possibilities to meet with other MSM?

*Probe*

- *Can you plan a meeting with other MSM at a public place like at the city gardens ad talk for a while?*

**Health needs analysis**

Let us discuss bout your health needs of the men who have sex with men in Tanga.

Can you tell me about the crucial needs that you and other men who have sex with men need?

*Probe*

- *If we want to establish a special health care to you what areas do you think should be covered to ensure good health to all?*

What are the other health needs that you personally need?

*Probe*

- *What do you think that you need but you are not getting it in the health sector just because you are MSM?*

Imagine that you are given a task to prepare a program to deal with improving health care towards men who have sex with men, how would you like it to be?

Probe

- How would you like the doctors to be like?
- Would you like to get things like seminars?
- Would you like this program to be known to the public?

If we decide to educate health workers about provision of friendly services towards MSM, what would you like us to teach them?

What role do you think the use of Internet and mobile phones can play in helping you to improving your healthcare?
