# Supplementary material for: Acting within an increasingly confined space: A qualitative study of sexual behaviours and healthcare needs among men who have sex with men in a provincial Tanzanian city
Source: PLoS One. 2017 Aug 17;12(8):e0183265. doi: 10.1371/journal.pone.0183265 (PMC5560662; doi:10.1371/journal.pone.0183265)
Supplement: S2 Text — (DOCX) [file pone.0183265.s002.docx]

**S2 Thematic Interview Guide- Swahili**

**Fomu ya Ridhaa-Soma kwa sauti**

**Mwonngozo wa mahojiano**

Jina langu ni ____. Kwa sasa tunafanya utafiti juu ya mitazamo na uzoefu wa maisha kati ya wanaume wanaojihusisha kimapenzi na wanaume hapa Tanga na tutaongelea vipengele vya mahusiano ya kimapenzi, tabia za kimapenzi, familia, mitazamo ya jamii na upatikanaji wa huduma za kiafya. Matokeo ya utafiti huu yatachangia uelewa juu ya mahitaji ya kiafya kwa wananume wanaojamiiana na wanaume nakujua ni jinsi gani itawezekana kuhusisha wanaume wananojamiiana na wanaume katika miradi mbalimbali inayotoa huduma za VVU na magonjwa mengine ya ngono.

Utafiti huu unafanyika kwa ushirikiano wa chuo kikuu cha Dar es Salaam na Chuo kikuu cha Texas, Marekani

Ushiriki wako katika utafiti huu ni wa hiyari na hutaulizwa au kulazimishwa kujibu maswali ambayo hutaki kujibu. Pia napenda kukuhakikishia kuwa taarifa zote zitakazokusanywa wakati wa mahojiano zitabaki kuwa siri. Jina lako halitaandikwa sehemuyotote katika karatasi zetu au machapisho yetu na pia haitawekwa kwa namna yoyote ambayo mtu anaweza kugundua ni nani aliyetoa taarifa hizi. Kwa vile hatutakutaja kwa namna yoyote ile tunakuhakikishia kuwa hakuna madhara yoyote yatokanayo na ushiriki wako katika utafiti huu.

Hata hivyo, taarifa utakazotoa zitatumika kutengeneza machapisho mbalimbali au majadiliano ambayo yanaweza kutumika katika kuongeza ujuzi wa VVU/UKIMWI na magonjwa mengine ya zinaa. Hata hivyo katika machapisho haya jina lako wala taarifa zako binafsi hazitaandikwa popote kwa namna ambayo itaonesha ni nani aliyetoa taarifa hizi.

Mahojiano yetu yatachukua wastani wa saa moja na nitarekodi mazungumzo yetu kwenye chombo maalum. Mahojiano haya yatafutwa mara baada ya kukamilisha zoezi la kusikiliza na kuandika pointi mbalimbali ulizoongea wakati wa mahojiano.

Kama utakuwa na maswali yoyote yale, ushauri au dukuduku kuhusuana na utafiti huu tafadhali usisite kuwasiliana nasi kwa anuani zilizopo katika kadi nitakayokupatia. Tafadhali jisikie huru kuwasiliana nasi na tutakupatia msaada (toa kadi yenye namba za mawasiliano)

Je una maswali yoyote?

**Unakubali kushiriki katika mahojiano haya? Sema “Ndiyo” au “Hapana” na Tarehe ya leo.**

**HOJAJI YA KISWAHILI**

Tutaongelea Zaidi kuhusu mambo mbalimbali yanayowakabili wanaume wanaojamiiana na wanaume hapa Tanga. Tafadhali jisiskie huru.

***Historia ya kimapenzi***

Tafadhali tuongelee kuhusu historia yako ya kimapenzi. Kumbuka kuwa kila kitu tunachoongelea hapa kitabaki kuwa siri

Ni lini uligundua kuwa unavutiwa kimapenzi na wanaume wengine?

*Dodosa:*

- *Unakumbuka ni nini hasa kilitokea ukagundua kuwa unavutiwa kimapenzi na wanaume wengine?*
- *Ulikuwa unaishi na nani wakati huu?*

Ulikuwa na umri gani ulipojamiiana kwa mara ya kwanza?

*Dodosa:*

- *Ilikuwa mwananume au mwananmke?*

Tafadhali niambie kuhusu mwanaume wa kwanza kuwa naye kimapenzi, mlikutana wapi?

Alikuwaje? Alikuwa mkubwa au mdogo kiumri kwako?

*Dodosa:*

- *Ulijisikiaje kuhusu tofauti ya umri?*

Wakati mwingine tunajikuta tunalazimishwa kukutana kimapenzi na watu ambao hatutaki au kinyume na ridhaa zetu. Najua hili swali ni gumu kwa kiasi Fulani lakini ningependa kujua kama hii hali imewahi kukuta.

*Dodosa:*

- *Kama ndio, ilikuwaje?*
- *Ilikuwa mtu mwenye umri mkubwa kuliko wewe?*

**Mahusiano yako ya kimapenzi kwa sasa**

Mahusiano yako na wanawake yakoje?

*Dodosa:*

- *Una mpenzi wa kike au unao Zaidi ya mmoja?*
- *Kama ndio umekuwa nae kwa muda gani?*
- *Anajua kuwa unajamiiana na wanaume?*

Umewahi kuwa na wapenzi wananawake katika maisha yako?

Nini hasa kinakuvutia au kilikuvutia kuwa nao kimapenzi?

Kwa sasa ni jinsia gani inakuvutia Zaidi kimapenzi?

Tafadhali niambie kuhusu hali yako ya kimapenzi kwa sasa.

*Dodosa:*

- *Una wapenzi wangapi kwa sasa?*
- *Je ni wapenzi wa kudumu au wapenzi wa kibiashara?*
- *Ni sehemu gani huwa unakutana na wapenzi hawa kwa ajili ya kufanya mapenzi?*
- *Kwanini unapendelea sehemu hizi?*

Unafanya mapenzi mara ngapi kwa wiki?

Ni vitu gani vinasababisha ufanye mapezi na mwenzi au wenzi wako?

*Dodosa:*

- *Pombe ina nafasi gani wakati wa kufanya mapenzi?*
- *Madawa ya kulevya yana nafasi gani wakati wa kufanya mapenzi?*
- *Mazingira mfano sehemu zenye mvuto au sehemu za kujificha zina nafasi gani wakati wa kufanya mapenzi?*

Simu ya mkononi ina nafasi gani katika maisha yako ya kimapenzi?

*Dodosa:*

*- Je unaitumia kuwasiliana na mpenzi wako?*

**Maisha ya kijamii**

Unaweza kuniambia marafiki wana nafasi gani katika maisha yako?

*Dodosa:*

- *Ni kwa kiasi gani marafiki zako wana nafasi katika kufanya maamuzi kiatika maeneo mbalimbali ya maisha yako?*
- *Ni kwa kiasi gani marafiki zako wana nafasi katika maisha yako ya kimapenzi?Mfano ukipata mpenzi marafiki zako wana nafasi ya kukuambia usiwe nau au usiwe nae?*
- *Ni ushauri gani marafiki hawawezi kukupa na kwa nini?*

Kwa uzoefu wako ni kwa kiasi gani marafiki wanachangia katika kupata mpezi?

*Dodosa:*

- *Je marafiki wanaweza kukuambia ufanye au usifanye kitu Fulani?*

Watu waliokuzidi umri wana nafasi gani katika maisha yako?

*Dodosa:*

- *Je watu waliokuzidi umri wana ushawishi wowote katika maisha yako? Mfano wanaweza kukushauri katika maisha yako ya kimapenzi?*

Familia yako ina nafasi gani katika maisha yako?

*Dodosa:*

- *Je wazazi wako a ndugu zako wanajua kuwa unafanya mapenzi na wanaume?*
- *Kama hapana kwa nini hujawaambia?*
- *Kama ndio, unadhani kwa nini wamekubali hii hali?*

**Unyanyapaa**

Tumekuwa tukiongelea kuhusu maisha binafsi na sasa naomba tuongelee kuhusu unyanyapaa. Hali ya unyanyapaa ipoje hapa katika jiji la Tanga?

*Dodosa*

- *Niambie Zaidi kuhusu unyanyapaa, unaoneshwaje?*
- *Jamii ya watu wasojihusisha na mapenzi ya jinsia moja wanakuonaje?*

Unyanyapaa unaoneshwaje katika familia?

Unyanyapaa unaoneshwaje katika maeneo ya kazi?

*Dodosa:*

- *Umeajiriwa?*
- *Itakuwaje endapo wafanyakazi wenzio watagundua kuwa wewe ni shoga?*

Ukipata tatizo linalokufanya uende kituo cha polisi inakuwaje? Mfano ukiibiwa vitu ukaenda kuripoti polisi unapata msaada kama wengine?

Je kuna unyanyapaa wowowte unaofanyiwa pale unapohitaji huduma za kijamii mfano kukopa hela kwa mtu au kuhudumiwa unapoumwa?

Mfano ukimuona mwanaumme mzuri ukampeda unaweza kumtongoza?

*Dodosa:*

- *Unatumia mbinu gani?*
- *Unadhani ukimtongoza moja kwa moja atakunyanyapaa?*

Vipi kuhusu unyanyapaa wa wazi wazi unaweza kuniambia uzoefu wako upoje katika hili?

*Dodosa:*

- *Umewahi kunyanyapaliwa hadharani kwa kuwa wewe ni mwanaume unayejamiiana na wananume?*

Unyapaa unakufanya ujisikieje hasa pale unapokutokea wewe au maafiki wa karibu?

Kama sheria ingebadilika leo na kuruhusu mapenzi ya jinsia moja unadhani nini kingebadilika katika maisha yako?

**Unyanyapaa katika sekta ya afya**

Naomba uniambie kuhusu unyanyapaaa unaowakuta wanaume wanaojamiiana na wanaume katika vituo vya afya

*Dodosa*

- *Unyanyapaa unaoneshwaje huko?*
- *Wagonjwa wenzako huwa wanakuchukuliaje?*
- *Wahudumu wa afya, manesi na madaktari wanakuchukuliaje?*
- *Unaweza kuniambia uzoefu wako binafsi katika hili?*

Ni kwa kiasi gani unyanyapaa unafanya ushindwe kupata vipimo na matinbabu ya magonjwa ya zinaa?

*Dodosa:*

- *Unadhani kuna changamoto gani unapata katika huduma za afya?*
- *Unadhani marafiki zako wanapata changamoto gani katika huduma za afya?*

Unadhani unyanyapaa unachangia vipi katika kufanya mapenzi yasiyo salama na kutumia madawa ya kulevya?

Unadhani unyanyapaa una nafasi gani katika kusababisa matibabu binafsi?Yani pale unaponunua dawa kutoka katika duka la dawa na kujitibu bila kupata ushauri wa daktari?

**Tathmini ya huduma za afya:**

Umewahi kupata magonwa yanayoambukizwa kwa njia ya kujamiiana?

*Dodosa:*

*- Kama ndio, unaweza kuniambia dalili zake?*

Ulipopata hili tatizo ulienda wapi kupata matibabu? (Kama jibu ni ndio)

*Dodosa:*

- *Kwa nini ulichagua sehemu hii?*
- *Niambie zaidi kuhusiana na wewe kwenda sehemu hiyo, nini kilitokea?*

Umewahi kupima virusi vya UKIMWI?

*Dodosa?*

- *Kwa nini bado hujapima?(kama bado)*
- *Ulienda wapi kupima na kwa nini ulienda hapo (kama ndio)*
- *Uzoefu wako ulikuwaje?(Kama ndio)*

**Tabia Hatarishi**

Tuongelee kuhusu mambo yanayohusiana na tabia hatarishi. Untathmini vipi kama tabia Fulani ni hatarishi na nyingine sio?

*Dodosa:*

- *Unatambuaje kama tabia fulani ni hatarishi?*
- *Unafanyaje maamuzi kuhusu tambia mbalimbali zinazoweza kukufanya upate virusi vya UKIMWI na magonjwa mengine ya zinaa?*

Sasa niambie unajuaje kuwa mpenzi wako yupo kwenye hatari ya kupata maambukizi ya virusi vya UKIMWI na magonywa ya zinaa?

*Dodosa:*

- *Kwa mfano unajuaje kama mpenzi wako ana wapezni wengi kitu ambacho kinakuweka katika hatari ya kupata maambukizi ya virusi vya UKIMWI?*
- *Unafanyaje pale ambapo mpenzi wako anakataa kutumia kondomu?*

Kuna madhara gani ya unyanyapaa pale unapotafuta mpeniz?

Unaweza kuniambia ni kwa kiasi gani unyanyapaa unaathiri mawasiliano yako na mweza wako?

*Dodosa:*

*- Je kuna vitu ambavyo huwezi kuzungumza nae?*

Je unaweza kuniambia ni kwa kiasi gani unyanyapaa unaathiri uwezwkano wa wewe kukutana na wanaume wengine wanaojamiiana na wanaume?

*Dodosa:*

- *Unaweza kupanga kukutana na mwanaume mwingine anajemiiana na mwananume kwenye sehemu za wazi mfano bustani za umma na kuongea kidogo?*

**Tahmini ya mahitaji ya kiafya**

Tuongelee kuhusu mahitaji ya kiafya ambayo wanaume wanaojamiiana na wanaume hapa Tanga wanayo.

*Dodosa:*

- *Kama tukitaka kuweke kliniki maalumu kwa ajili ya kutoa huduma za afya kwa wanaume wanaojamiina na wanaume unadhani ni vitu gani lazima tuweke ili kutoa huduma bora kwa wote??*

Je, una mahitaji gani binafsi ya kiafya?

*Dodosa:*

- *Unadhani wewe kama mwananume unayejemiiana na wanaume una mahitaji gani muhimu ambayo sekta ya afya haikupatii?*

Kama ungepewa kazi ya kuandaa mchakato wa kuboresha huduma za afya kwa wanaume wanaojamiiana na wanaume ungependa mradi huo uweje?

Dodosa:

- Ungependa madaktari waweje?
- Ungependa kupata semina?
- Ungependa huduma hizi zijulikane kwa jamii?

Kama tukitaka kuelimisha wahudumu wa afya kuhusiana na kutoa huduma rafiki kwa wanaume wanaojamiiana na wanaume ungependa tuwaelimishe kuhusu nini hasa?

Unadhani matumizi ya intaneti na simu za mikononi yana nafasi gani katika kusaidia kuboresha utoaji wa huduma za afya?
